# Evaluation of a low-resource soy protein production method and its products

**DOI:** 10.3389/fnut.2023.1067621

**Published:** 2023-04-20

**Authors:** Ece Gulkirpik, Annette Donnelly, Kephas Nowakunda, Keshun Liu, Juan E. Andrade Laborde

**Affiliations:** ^1^Department of Food Science and Human Nutrition, University of Illinois at Urbana-Champaign, Urbana, IL, United States; ^2^Department of Agricultural and Consumer Economics, University of Illinois at Urbana-Champaign, Urbana, IL, United States; ^3^National Agricultural Research Laboratories, Kampala, Uganda; ^4^U.S. Department of Agriculture-Agricultural Research Service (USDA-ARS), National Small Grains and Potato Germplasm Research Unit, Aberdeen, ID, United States; ^5^Food Science and Human Nutrition Department, University of Florida, Gainesville, FL, United States

**Keywords:** soy protein concentrate, soy cake, malnutrition, green processing, sustainability, lipid oxidation, protein digestion

## Abstract

**Introduction:**

One key approach to achieve zero hunger in Sub-Saharan Africa (SSA) is to develop sustainable, affordable, and green technologies to process nutritious food products from locally available sources. Soybeans are an inexpensive source of high-quality protein that may help reduce undernutrition, but it is underutilized for human consumption. This research evaluated the feasibility of a low-cost method developed initially at the United States Department of Agriculture to produce soy protein concentrate (SPC) from mechanically pressed soy cake and thus create a more valuable ingredient to improve protein intake in SSA.

**Methods:**

The method was initially tested in the bench scale to assess process parameters. Raw ingredients comprised defatted soy flour (DSF), defatted toasted soy flour (DTSF), low-fat soy flour 1 (LFSF1; 8% oil), and LFSF2 (13% oil). Flours were mixed with water (1:10 w/v) at two temperatures (22 or 60°C) for two durations (30 or 60 min). After centrifugation, supernatants were decanted, and pellets were dried at 60°C for 2.5 h. Larger batches (350 g) of LFSF1 were used to examine the scalability of this method. At this level, protein, oil, crude fiber, ash, and phytic acid contents were measured. Thiobarbituric acid reactive substances (TBARS), hexanal concentration and peroxide value were measured in SPC and oil to evaluate oxidative status. Amino acid profiles, *in vitro* protein digestibility, and protein digestibility corrected amino acid score (PDCAAS) were determined to assess protein quality.

**Results:**

Bench scale results showed accumulation of protein (1.5-fold higher) and reduction of oxidative markers and phytic acid to almost half their initial values. Similarly, the large-scale production trials showed high batch-to-batch replicability and 1.3-fold protein increase from initial material (48%). The SPC also showed reductions in peroxide value (53%), TBARS (75%), and hexanal (32%) from the starting material. SPC’s *in vitro* protein digestibility was higher than the starting material.

**Conclusion:**

The proposed low-resource method results in an SPC with improved nutritional quality, higher oxidative stability, and lower antinutrient content, which enhances its use in food-to-food fortification for human consumption and is thus amenable to address protein quantity and quality gaps among vulnerable populations in SSA.

## 1. Introduction

Chronic undernutrition in infants manifests as stunting, which retards linear growth and neurocognitive development and is measured as two standard deviations below the height-for-age median of the WHO Child Growth Standards for children under five (1). Today, over 149 million children under the age of five globally are stunted, with most of the prevalence in Sub-Saharan Africa (SSA) and South Asia (2, 3). The United Nation’s Sustainable Development Goal 2 (SDG2) calls for zero hunger by 2030. To reach this goal, stunting, as followed in SDG 2.2.1, is targeted to decline 40% by 2025. As a result of work toward the SDG2, rates of stunting have fallen globally from a prevalence of 33% in 2000 to 22% in 2020 (3). The SSA region, however, is growing its share of the global total number of stunted children, with a 41% prevalence in 2020 (4). In Africa, stunting and undernutrition is a multi-generational issue (5). Regardless of crossing the stunting statistical cut-off, low BMIs in children impairs growth, hinders cognitive development, and leads to educational and adult work under performance (1, 6). The effects of undernutrition on neurocognitive development during infancy and childhood affect economic productivity into adulthood (7).

Diet quality, especially digestible protein, is shown to be a critical factor in stunting and wasting, particularly in SSA, where most of the energy comes from starchy staple foods, such as maize (8). Chronic protein malnutrition affects the ongoing development of higher cognitive process throughout childhood and can result in long-lasting impairment (9). Despite their low protein and micronutrient content, cereal-based complementary foods and porridges for young children remain critical sources of nutrients for a vast majority of infants and young school-age children in low- and middle-income countries, where the availability and access to animal-source foods and commercially fortified complementary foods and porridges are unattainable for many households (10–12). As a result, global recommendations have called for the development of cost-effective nutrient-dense foods for weaning infants and school-age children. Emerging research suggests nutrition interventions can ameliorate the effects of early childhood stunting (13). One pragmatic strategy is to combine two or three ingredients such as cereals, tubers, and legumes to complement protein and enhance nutrient density; this strategy is referred to as food-to-food fortification.

Among legumes, soybeans are a good source of quality protein and are high in fatty acids, minerals, and fat-soluble vitamins (14). Soy protein products are versatile and easily incorporated into blended foods for complementary feeding (15). The great majority of the world’s soybean production is crushed into oil and defatted meal by a solvent extraction process. While oil is refined for edible applications, defatted meal is mostly used for animal feed. Only a small portion of defatted meal is processed into soy flour, soy protein concentrate (>60–70% protein), and soy protein isolate (>90% protein) as food ingredients (16). Alternatively, soybeans can be mechanically pressed or extruded and expelled to produce oil and soy cake (17). Soy cake still contains 6–13% oil, depending on actual processing parameters, and can be processed into low-fat soy flour.

In the SSA region, soybeans represent a small (15%) but growing fraction of oilseeds (26.2 million mt) grown for human consumption (18). According to the U.S. Department of Agriculture Foreign Agricultural Service (USDA FAS), the annual production of soybean oil in South Africa, the largest soybean oil producer in SSA, is forecasted at 270,000 MT for 2022, which together with import represents ~24% of the 1.4 million MT of edible oil consumed in South Africa (19). As soybeans have a lower oil content than other oilseeds, solvent extraction is the preferred method. Nonetheless, the significant capital expense of solvent extraction makes it unreachable for most processors in SSA, leaving mechanical oil extraction as the most prevalent method of extraction. Although soy cake can be further processed into higher value products, it has been underutilized for human consumption in this region. Therefore, there is a need for alternative approaches to add value to this highly nutritious low-fat by-product, which can then be used for food-to-food fortification approaches to enhance the protein contents of staple meals in SSA.

Soy protein concentrate (SPC) is a nutrient-dense and highly functional soybean product. It has been widely used to add favorable functionalities to a wide variety of food and feed products at a reduced cost (20) and has played a significant role in human nutrition, especially in low-income settings; therefore, it can help alleviate malnutrition in SSA (21, 22). There are three current methods for preparing SPC: acid leaching (isoelectric pH 4.5), aqueous alcohol (60–80%) extraction, and heat denaturation/hot water leaching. They all share the same principle by insolubilizing proteins and complex carbohydrates and removing soluble carbohydrates. (23). SPC can serve as a superior food ingredient owing to its high-quality protein and fiber content (24). Most African countries, however, have an underdeveloped food sector. There are few large-scale facilities for processing defatted soy meal into protein concentrates. Moreover, the high costs of imported protein concentrates make it unfeasible to support any recommendations for increased incorporation of these ingredients into foods for human consumption (25). Because of these factors, the limited utilization of SPC in SSA is mainly due to the inadequate processing capabilities, high processing costs, and complexity of current processing techniques.

Soybeans contain several anti-nutrients, most of which are reduced to some degree after protein extraction. The anti-nutritional factors, such as trypsin inhibitors, lectins, phytates, saponins, and oligosaccharides, limit the utilization of soybeans for human and animal nutrition. Phytic acid, for example, makes up more than half of the total phosphorus in soybeans and can reduce the absorption of essential micronutrients such as Ca, Fe, and Zn (16). Additionally, the presence of isoflavones in soybeans and their potential phytoestrogenic effects might be of concern. Though isoflavones have shown phytoestroenic effects under specific conditions in humans and animals, the consumption of soybean diets has been more associated with protection against certain estrogen-depended cancers than their promotion (26). A recent study showed no hormonal effects after long-term feeding of soy protein-containing formulas in infants (27). In the SSA region, soy-fortified cereals for children and for adults are present in most supermarkets.

Recently, a less expensive technique to prepare SPC was first proposed by Liu and his colleagues at the USDA, which uses limited resources and water as a solvent (28). This process eliminates the use of solvents and acids and is amenable to the use of low-fat soy flour as the starting material. The rendered products from this novel technique had not been characterized in terms of protein quality and oxidative stability. These are important considerations to promote the use of SPC, especially from low-fat soy flour obtained from mechanical oil pressing, which might affect these characteristics due to the high temperature and pressure applied. The current study was aimed at evaluating the alternative low-resource SPC production method with low-fat soy cake as a starting material, by characterizing SPC in terms of protein yield and quality, proximate composition, phytic acid content, and oxidative stability. The hypothesis was that the application of this water-leaching technique will result in SPC with improved protein quality (in terms of content and digestibility) and oxidative stability, and reduced antinutrient content. The alternative processing approach was first implemented on a bench scale using four different starting materials. Considering the results obtained from the bench-scale trials, the alternative processing method was then tested on a larger scale, in which three separate batches of SPC were produced from the same initial material.

## 2. Materials and methods

### 2.1. Raw materials and chemicals

Four different soy flour were used as raw materials. Defatted soy flour (DSF) was purchased from Archer Daniels Midland Company (Baker’s Soy Flour with min 53% protein and max 3% fat, Decatur, IL, United States). Defatted toasted soy flour (DFTS) was made by toasting the DSF at 109 ± 11.22°C for 30 min. Two different extruded expelled low-fat soy flours (LFSF1 and LFSF2 with 8 and 13% oil content, respectively) were provided by Tiger Soy LLC (Mexico, MO, United States). These two materials were produced by drying and grinding the soy cake, which was obtained after the extrusion and expelling process of soybeans. All other chemicals were of analytical grade or purer and acquired from Fisher Chemical Company (Hampton, NH, US) or Sigma Aldrich (St. Louis, MO, United States).

### 2.2. Low resource SPC production method

#### 2.2.1. Bench scale production process

Defatted or low-fat soybean flour samples (3.00 ± 0.01 g) and distilled water (1:10 w/v) were added into a 50 ml centrifuge tube and mixed in a shaking incubator (Incu-Shaker Mini, Benchmark Scientific; Sayreville, NJ, United States) at 250 rpm, at two different temperatures (22 and 60°C) for two different durations (30 and 60 min). In the first trial, the 30 min duration was tested at 22°C and 60°C, respectively. Because the increased extraction temperature did not increase SPC protein content substantially, not to mention of its higher energy consumption, for the 60 min extraction, only 22°C was examined in the next trial. The slurry was then centrifuged at 3,810 × *g* for 30 min at room temperature (Sorvall™ ST 8, Thermo Scientific™; Waltham, MA, United States). After the solid–liquid separation, the precipitate (aka wet protein concentrate) was transferred into a pre-weighed drying pan for drying in a conventional lab oven (Heratherm™, Thermo Scientific™, Waltham, MA United States) at 60°C for 2.5 h. Dried SPC (contained 10–15% moisture) was ground into powder (to pass 100 μm openings) using a coffee grinder (Hamilton Beach Inc.; Southern Pines, NC, United States). Ground SPC samples and raw materials were kept at-20°C until further analysis.

#### 2.2.2. Large scale production process

Three separate batches of SPC were produced from LFSF1 by following the previously described production process with some modifications. For making each batch, 350 g of LFSF1 and 3.5 l of distilled water were mixed using a mixer (KitchenAid®; Benton Harbor, MI, United States) for 30 min at 22°C. After the washing process with water, the mixture was transferred into a 5 l plastic container, and it was covered with aluminum foil. For solid–liquid separation, the mixture was first left for settlement for 2 h at room temperature. At the end of 2 h, the precipitate was separated by using a woven 200-micron commercial-grade nut-milk bag (Nut Milk Bag; Vandoona LLC., NY, United States). The wet protein concentrate collected in the bag was then uniformly spread onto a pre-weighed metal drying tray and dried by using a dehydrator (Excalibur Dehydrators®, Sacramento, CA, United States) at 60°C for about 4 h until its moisture content decreased below 15%. The dried SPC was then ground into a powder (to pass 150 μm openings) using a hammer mill (Lab Mill 3,100, PerkinElmer Inc.; Waltham, MA, United States). Dried SPC materials were placed in sealed bags and kept at −20°C until further analysis.

### 2.3. Determination of total protein content

The total protein content of raw materials and their SPCs produced at the bench scale trials were determined by using the Coomassie Plus (Bradford) Assay (Thermo Scientific; Rockford, IL, USA) kit after the protein extraction. The method of Mujoo et al. (29) as described in (30) was followed for protein extraction with some modifications. The samples were defatted prior to protein extraction using hexane. Raw materials or SPC were extracted with hexane (1:3 w/v) twice using a shaking incubator at 250 rpm for 1 h after vortexing for 20 s at room temperature. The mixture was then centrifuged at 1,663 × *g* for 5 min at room temperature, and the supernatant was transferred into a new centrifuge tube. The defatted wet precipitant was dried overnight at room temperature under a chemical hood. A 0.5 g of dried defatted sample and a 15 mL solution containing 30 mM Tris buffer at pH 8.0 and 10 mM b-mercaptoethanol were first sonicated by using a VC-750 ultrasound generator at 20 kHz and 40% amplitude for 2 min (Sonics & Materials, Inc.; Newtown, CT, United States) to reduce the particle size and increase the solubilization of proteins in the samples. The sonicated solution was then transferred into a centrifuge tube to be used in the estimation of protein concentration.

Following the Coomassie Plus (Bradford) Assay kit protocol, 10 μl of protein calibration standards and diluted samples were reacted with 300 μl of the Coomassie Plus Reagent in microplate wells. The microplates were incubated at room temperature for 10 min before the absorbance was read (595 nm) using a microplate reader (SpectraMax M2e, Molecular Devices; San Jose, CA, United States). According to the established calibration curve of BSA standards, the concentration of each sample was determined, and it was corrected based on the dilution factor and sample weight by using Eq. (1):


(1) $$\mathrm{Total Protein Concentration}\;\left(\frac{g\;\mathrm{protein}}{g\;\mathrm{sample}}\right)=\frac{x\times \mathrm{DF}\times V}{W\times 10^{6}}$$

where x was the concentration (μg/mL) calculated from the standard calibration curve; DF was the dilution factor, which was 20; V is the volume of Tris–HCl buffer used in protein extraction, which was 15 ml; W is the sample’s weight, which was 0.5 ± 0.05 g, and 10^6^ is the unit conversion factor for μg to g.

The total protein content of LFSF1 and its SPC produced at the larger scale trials were estimated by quantifying their nitrogen content (%) of samples using Exeter Analytical – Model CE440 CHN analyzer. Nitrogen was converted to protein by multiplying it with 6.25 conversion factor. Results were displayed on a dry matter basis by using the moisture content of each sample.

### 2.4. Determination of SPC production yield and protein recovery

The SPC production yield was calculated using Eq. (2) (31), while the protein recovery was calculated according to Eq. (3) (32),


(2) $$\mathrm{SPC}\;\mathrm{Production Yield}\;\left(\%\right)=\frac{\mathrm{Weight of Dried}\;\mathrm{SPC}}{\mathrm{Weight of}\;\mathrm{Raw}\;\mathrm{Material}}\times 100$$

(3) $$\mathrm{Protein recovery}\left(\%\right)=\frac{\mathrm{Mass of Protein in}\;\mathrm{SPC}}{\mathrm{Mass of Protein in}\;\mathrm{Raw}\;\mathrm{Material}}\times 100$$

### 2.5. Determination of oil content

The crude oil content of raw materials and their SPCs used in the bench-scale production trials were determined as described by Yue et al. (33) with some modifications. Samples were extracted with hexane (1:3 w/v) in a centrifuge tube using a shaking incubator at 250 rpm for an hour at room temperature after vortexing for about 20 s. The mixture was then centrifuged at 1,663 × *g* for 5 min at room temperature, and the supernatant was transferred into a new tube. The extraction process was repeated three times and the supernatant was collected and added to the previous one. Hexane was then evaporated from the collected supernatant by using a nitrogen evaporator (Organomation Associates; Berlin, MA, United States). The amount of oil extracted from the sample was determined by calculating the weight change in the tube holding supernatant after the solvent evaporation, and it was presented in % d.b. unit by using the moisture content of samples.

To estimate oil contents of samples tested in the large-scale production trials, the Folch et al. (34) described in the study of Wu and Wang (35) was followed with some modifications. Samples of about 24 g were weighted in a 250 ml glass beaker. A chloroform/methanol solution (2:1 v/v) was prepared and added to the beaker at a solvent to sample ratio of 5:1. The mixture was stirred using a magnetic stirrer on a stirring plate (Denville Scientific Inc.; Holliston, MA, United States) for 1 h at room temperature. The mixture was then filtered using a filter paper (Whatman® #1) and the filtered solution was collected into a separating funnel. The crude extract in the separating funnel was then mixed with 0.2 its volume of DI water and the mixture was allowed to separate into two phases by standing. The bottom layer was then collected in a pre-weight round bottom flask and the solvent was evaporated at room temperature using a rotary evaporator (Rotavapor® Model: R-200, Buchi Corporation; New Castle, DE, United States). The oil sample left in the flask was transferred into a pre-weighed, new centrifuge tube by washing it with chloroform which was then evaporated by using a nitrogen evaporator (Organomation Associates; Berlin, MA, USA). Total lipid content was determined gravimetrically after complete evaporation of chloroform in the oil sample, and it was presented in % d.b. units after correcting for moisture content.

### 2.6. Determination of moisture, ash, crude fiber, and total carbohydrates contents

The moisture contents (MC) of raw materials and SPCs were measured by a moisture meter (HB 43-SE Moisture Analyzer, Mettler Toledo, Switzerland). Crude fiber content of samples was determined by filter bag technique utilizing H_2_SO_4_ and NaOH digestion according to AOCS Ba 6a-05 method (36). Ash content was assessed following AOCS Ba 5a-49 protocol (37). Total carbohydrate content was determined from the difference of other determinations including total protein, moisture content, ash, and crude fat from the total dry matter (100%) as shown in Eq. (4) (38):


(4) $$\%\mathrm{Total carbohydrate}=100-\left(\begin{array}{l} \%\mathrm{moisture content}+\%\mathrm{total}\;\mathrm{ash}\\ +\%\mathrm{crude}\;\mathrm{fat}+\%\mathrm{crude protein} \end{array}\right)$$

### 2.7. Determination of phytic acid concentration

Phytic acid was determined using Phytic Acid Assay Kit (Megazyme Ltd.; Chicago, IL, USA) by following the instructions given in the kit and its published method (39). This method is based on the use of phytase and alkaline phosphatase to release all bound phosphorous, which is followed by the reaction of inorganic phosphorus with ammonium molybdate to form 12-molybdophosphoric acid. This is subsequently reduced under acidic conditions to molybdenum blue. Total inorganic phosphorus was estimated from the subtraction of before and after enzymatic hydrolysis. The phytic acid content was then calculated by using Eq. (5) in which 0.282 is the factor used to convert the measured phosphorus content to phytic acid content. Phytic acid contains 28.2% phosphorus, and the calculation assumes that the amount of phosphorus measured is explicitly released from phytic acid, not from any other sources.


(5) $$\mathrm{Phytic acid}\;\left(g/100g\right)=\frac{\mathrm{Phosphorus}\;\left(g/100g\right)}{0.282}$$

### 2.8. Determination of peroxide value

The peroxide value (PV) of raw materials and SPC samples was determined according to the AOCS (40). Extracted oil (1.00 ± 0.1 g) was accurately weighed into an Erlenmeyer flask and dissolved in 25 ml acetic acid and chloroform solution (3:2 v/v). 1 ml of saturated KI solution was added, and the mixture was left in the dark for 10 min. After that, 5 ml of starch solution (1% w/v) was added, and the color of the sample turned from orange to dark blue. Titration was conducted with 0.01 N sodium thiosulfate by vigorous shaking until the dark-blue color disappeared and the color of the sample became white and cloudy. A blank sample was also prepared by using water. Peroxide value was expressed as milliequivalent (mEq) of peroxide per kg oil, and calculated using Eq. (6):


(6) $$\mathrm{PV}\;\left(\mathrm{mEq}/\mathrm{kg}\right)=\frac{\left(V_{s}-V_{b}\right)\times N\times 1000\;}{W}$$

where *V_s_* is the volume of 0.01 N Na_2_SO_3_ consumed for a sample during titration (mL); V_b_ the volume of 0.01 N Na_2_SO_3_ used for blank during titration (mL); N is the normality of Na_2_SO_3_ solution (0.1 N), W is the weight of oil sample (g).

### 2.9. Determination of malondialdehyde concentration

The concentration of MDA in raw materials and SPCs was determined spectrophotometrically using the thiobarbituric acid reactive substances (TBARS) test as described in the study of Papastergiadis et al. (41) with some modifications. To extract MDA, a 2.4 ± 0.1 g of sample and a 15 ml solution containing 7.5% TCA (w/v) with 0.1% (w/v) of EDTA and 0.1% (w/v) propyl gallate were added into a 50 ml centrifuge tube and vortexed for 20 s. The mixture was then homogenized using a highspeed homogenizer (Polytron® Immersion Dispenser, Kinematica Inc.; Bohemia, NY, United States) at 18,000 rpm for 1 min. The homogenized sample was then filtered through a 150 mm filter paper (Whatman® #1) and the collected extract was used for MDA determination.

For the spectrophotometric determination of MDA, 2.5 ml of extract and 2.5 ml of TBA reagent (46 mM in 99% glacial acetic acid) were mixed in a 15 ml centrifuge tube and was heated in a dry bath incubator (Isotemp®, Fisher Scientific Co LLC; Pittsburg, PA, USA) at 100°C for 35 min. At the end of the heating period, the tubes were immediately placed in an ice bath. After cooling down to room temperature, the colored extracts were diluted with a trichloroacetic acid (TCA) solution (1:4 v/v) in separate tubes and vortexed. A 300 μl aliquot was transferred into a 96-well microplate wells and their absorbances were measured at 532 nm in a microplate reader. Standard solutions from MDA stock (1 mM) in 7.5% TCA were prepared from 1,1,3,3- tetraethoxypropane (TEP) and the calibration curve was prepared at a concentration ranging from 0.6 to 10 μM. To quantify the MDA concentration in samples Eq. (7) was used:


(7) $$\mathrm{MDA}\;\left(\frac{\mu g\;\mathrm{TEP}}{g}\right)=x\times 1\mu M\;\mathrm{TEP}\times \frac{V}{W}\times \mathrm{DF}$$

where x was obtained from the calibration curve; V is the volume (15 ml) of TCA solution used in the extraction; W is the sample weight and DF is the dilution factor (5).

### 2.10. Determination of hexanal concentration

The hexanal concentration of LFSF1 and its SPCs made in a larger scale was determined by headspace solid-phase micro-extraction using the gas chromatography, mass spectrometry (HS-SPME-GC–MS) technique described by Khrisanapant et al. (42) with some modifications. A 0.25 mg of sample was weighed into a 20 ml headspace vials (Restek™ Headspace Screw-Thread Vials, 18 mm, Fisher Scientific Co LLC; Pittsburg, PA, United States) and covered with magnetic screw caps with PTFE/silicone septa and kept at −18°C until the analysis. For identification of hexanal, a 5 μl internal standard (IS) solution containing 0.2 μg/μl of 2-methyl-3-heptanone was spiked in each sample vial. Then, vials were placed into a CombiPal autosampler (Leap Technologies, Inc.; For Lauderdale, FL, United States) connected to a GC–MS system (Agilent Technologies, Inc.) where they were incubated at 40°C for 5 min. Afterwards, a SPME fiber (1.5 cm, 50/30 μm DVB/Carboxen™/PDMS, StableFlex™) was inserted to the headspace of vials and extracted volatile compounds for 30 min at 40°C.

The extracted volatile compounds were released into the GC–MS system using hot spitless injection at 260°C for 4 min followed by 20 min post injection fiber conditioning in the inlet and injected to the Stabilwax® GC column (30 m × 0.25 mm id × 0.25 μm film thickness, Restek; Bellenfonte, PA, USA) for separation of volatile compounds with helium as the carrier gas at a 1 ml/min constant flow rate. The oven temperature of GC was arranged from 40°C to 225°C at 4°C/min with initial and final holding times of 5 and 30 min, respectively. The capillary direct interface temperature, ionization energy, mass range, electron multiplier voltage and scan rate conditions of the mass selective detector were 230°C, 70 eV, m/z35 to 500, Autotune +200 V and 50 scans/s, respectively [adapted from (43)].

With the help of NIST 14 library and Leco Chroma TOF software (version 4.51.6), volatile compounds were identified and the peak area for hexanal were found for each sample. To determine the hexanal concentration in each sample, Eq. (8) was used:


(8) $$\begin{array}{l} \mathrm{Hexanal Concentration}\;\left(\mathrm{ppm}\right)=\frac{\frac{{\mathrm{PA}}_{\mathrm{Hexanal}}}{{\mathrm{PA}}_{\mathrm{IS}}}}{\frac{W_{\mathrm{Sample}}}{W_{\mathrm{IS}}}}\times \mathrm{IS Concetration}\\ \;\;\;\;\;\;\;\;\;\;\;\;\;\;\;\;\;\;\;\;\;\;\;\;\;\;\;\;\;\;\;\;\;\;\;\;\;\;\;\;\;\;\;\;\;\;\;\;\;\;\;\;\;\;\;\;\;\;\;\;\;\;\;\;\;\;\;\;\;\;\;\;\;\;\;\;\;\;\;\times 1000\times \mathrm{Rf} \end{array}$$

Where PA_hexanal_ and PA_IS_ are peak area of hexanal and internal standard solution, respectively; Wsample is the mass of sample (250 μg) and WIS is the mass of internal standard spiked in each sample (0.2 μg/μl × 5 μl = 1 μg); concentration of IS was 0.2 μg/μl; 1,000 was the factor for unit conversion from μg/μl to ppm (mg/L) and Rf was the response factor for hexanal, which was obtained from previously plotted calibration curves for peak area ratio (hexanal:IS) versus mass ratio (hexanal:IS), and it was considered equal to 1.

### 2.11. *In-vitro* protein digestibility and protein digestibility corrected amino acid score

The amino acid (AA) concentrations of LFSF1 and its SPCs, except tryptophan and cysteine, at larger scale trials were determined using reverse phase HPLC with UV. Briefly, ground samples were hydrolyzed to single amino acids. After mixed with internal standards, the samples are dried in glass tubes in a vacuum concentrator and subjected to vapor phase hydrolysis using 6 N HCl at 150°C for 1.5 h under argon atmosphere in the presence of 2% phenol. Then, they are subsequently reconstituted in 0.4 N Borate Buffer to increase their pH to 10 and transferred to an autosampler (Agilent G1367E) for automated derivatization and loading. The derivatized amino acids were separated by reverse-phase HPLC (Agilent 1,260) and detected by UV absorbance (primaries at 338/390 nm and secondaries at 266/324 nm) with a variable wavelength detector (G1365D) using an in-line fluorescence detector (G1321B) and quantitated. The AA composition of the samples was expressed as g AA/100 g protein. Cysteine analyses were obtained following the AOAC Official Methods 982.30 E(b) (44), while tryptophan content was measured based on an enzymatic protocol described in Sessa, et al. (45) followed by a colorimetric determination described in Holz (46).

Digestion of LFSF1 or SPC samples for calculating the PDCAAS was carried out by following the protocol provided in the protein digestibility assay kit (K-PDCAAS, Megazyme Ltd.; Bray, Ireland). This assay is based on the simulated gastric and intestinal digestion of samples. After digestion, larger proteins are precipitated using TCA followed by centrifugation. Free amino acids are reacted with ninhydrin and respective purple development was followed at 570 nm in a plate reader and absorbances were compared against L-glycine as the standard for quantification. Furthermore, the amount of essential amino acid (EAA) for each of the samples (g/100 g protein) was calculated using the total protein (%) content as shown in Eq. (9):


(9) $$\mathrm{EAA}\;\left(g/100g\;\mathrm{protein}\right)=\frac{\mathrm{EAA}\;\left(g/100g\;\mathrm{sample}\right)}{\mathrm{Protein}\;\left(\%\right)}\;$$

The first limiting EAA in each sample was identified from the ratio of EAA (mg/g protein), also known as amino acid score (AAS), using the FAO/WHO recommended profile for pre-school children using Eq. (10). The EAA with the lowest ratio is the limiting amino acid. Finally, the *in vitro* PDCAAS values of LFSF1 and its SPCs were calculated by multiplying the *in vitro* digestibility by the limiting amino acid ratio.


(10) $$\mathrm{AAS}=\frac{\mathrm{Individual}\;\mathrm{AA}\;\mathrm{in}\;\mathrm{mg}/g\;\mathrm{protein in sample}}{\mathrm{Individual}\;\mathrm{AA}\;\mathrm{in}\;\mathrm{mg}/g\;\mathrm{protein in reference sample}\;}$$

### 2.12. Statistical analysis

For each sample, the above measurements were carried out in triplicates, except nitrogen, ash, fiber and amino acid profile analysis, which was done in duplicates. The collected data presented in the tables and figures are the mean values ± standard deviations (SD). Data were analyzed by using Microsoft Excel (Microsoft, WA, United States) and OriginPro 2021 (OriginLab Corporation, MA, United States), and it was subjected to One-way Analysis of Variance (ANOVA), except for the comparison for *in-vitro* protein digestibility and PDCAAS results of LFSF1 and its SPC for which paired Student’s *t*-test was employed. The Fisher’s Least Significant Difference test was used to compare means. Statistical significance was established at an alpha of 0.05.

## 3. Results

### 3.1. Protein and oil contents, oxidation level, and phytic acid concentration of SPC produced at the bench scale

**Figure 1:**
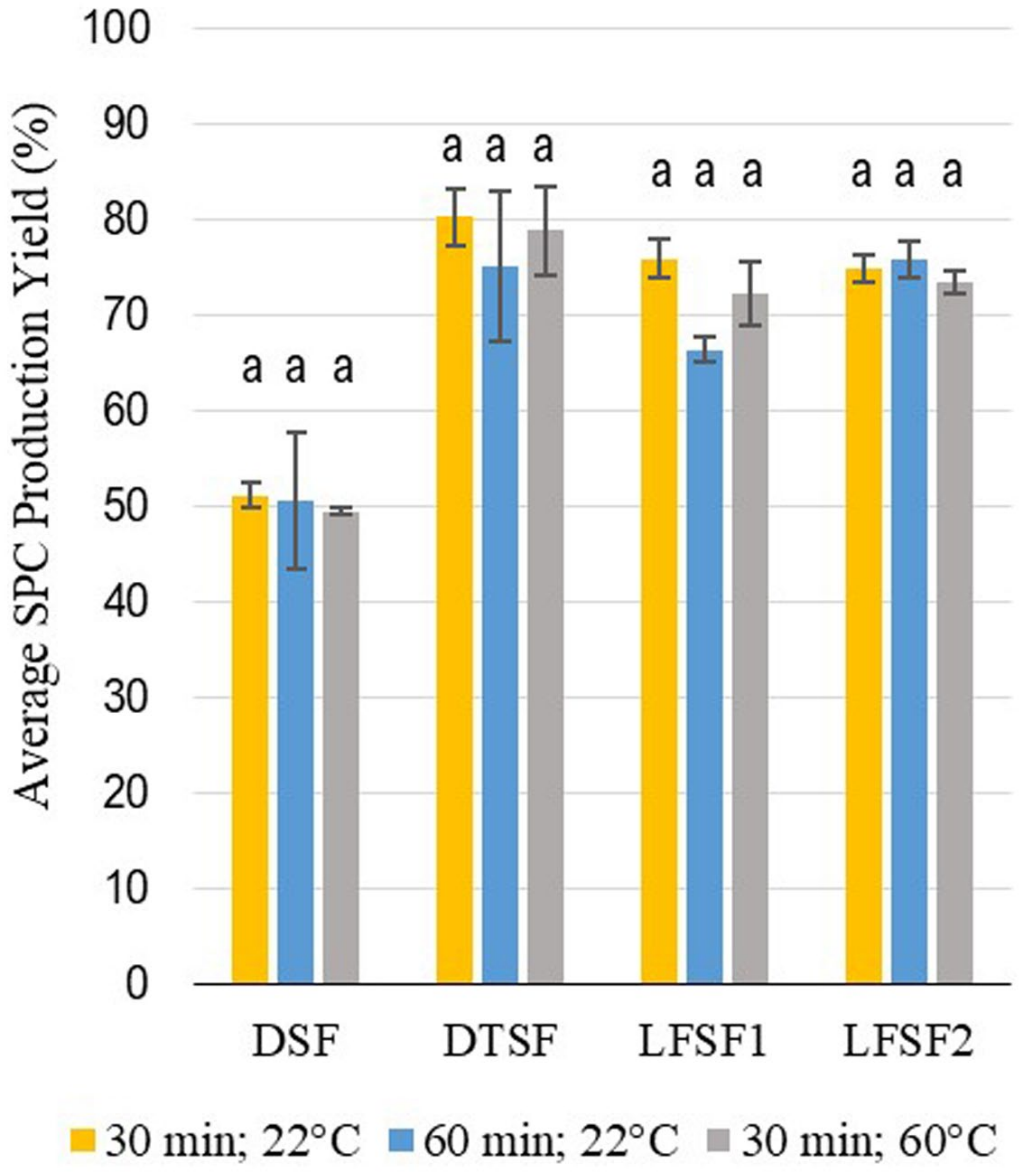
Average yields of SPC as prepared from defatted soy flour (DSF), defatted toasted soy flour (DTSF), low-fat soy flour 1 (LFSF1), and low-fat soy flour 2 (LFSF2) after different mixing times and temperatures. Bars represent means ± SD. Different superscript letters within each type of material group represent statistical differences (One-Way ANOVA and Fisher’s LSD test; *p* < 0.05).

The average total protein content of raw materials (DSF, DTSF, LFSF1, and LFSF2) and SPCs produced from them at the bench-scale trials applying different mixing parameters are given in Table 1. The starting materials, DFS, DTSF, LFSF1, and LFSF2, had total protein contents of 51.1 ± 2.0, 50.4 ± 0.7, 42.1 ± 2.3, and 41.8 ± 0.4% d.b., respectively. One of the major goals of the proposed production method was to create a low-cost, less resource-demanding alternative technique to produce SPC. As indicated in Table 1, increasing the mixing time or temperature does not impact the total protein content of SPCs produced (*p* > 0.05). Additionally, as shown in Figure 1, the average SPC production yields did not change in any of the samples with increasing mixing time and temperature (*p* > 0.05). Therefore, among the three combinations for the mixing process parameter tested, the least energy-demanding combination (30 min and 22°C) was selected, and SPCs produced by using these parameters were further investigated for other compositional properties.

**Table 1:** Total protein contents of DSF, DTFS, LFSF1, and LFSF-2 raw materials and their SPCs produced at the bench scale.

Raw material	Mixing process parameters		Total protein of SPCs (% d.b.)			
	Time (min)	Temperature (°C)				
DSF	Raw material		51.05	±	2.0	ab
	30	22	48.64	±	0.4	b
	60	22	50.67	±	0.8	ab
	30	60	51.38	±	0.6	a
DTSF	Raw material		50.35	±	0.8	b
	30	22	54.68	±	2.1	a
	60	22	54.41	±	0.0	a
	30	60	52.54	±	1.9	ab
LFSF1	Raw material		42.13	±	2.3	b
	30	22	63.70	±	0.4	a
	60	22	63.93	±	0.9	a
	30	60	62.56	±	2.0	a
LFSF-2	Raw material		41.83	±	0.4	b
	30	22	60.86	±	1.1	a
	60	22	61.32	±	1.3	a
	30	60	61.57	±	0.9	a

DSF, defatted soy flour; DTSF, defatted toasted soy flour; LFSF, low-fat soy flour; and SPC, soy protein concentrate. ^a,b^Means with a different superscript letter within the same sample type are statistically different from each other (*p* < 0.05).

**Figure 2:**
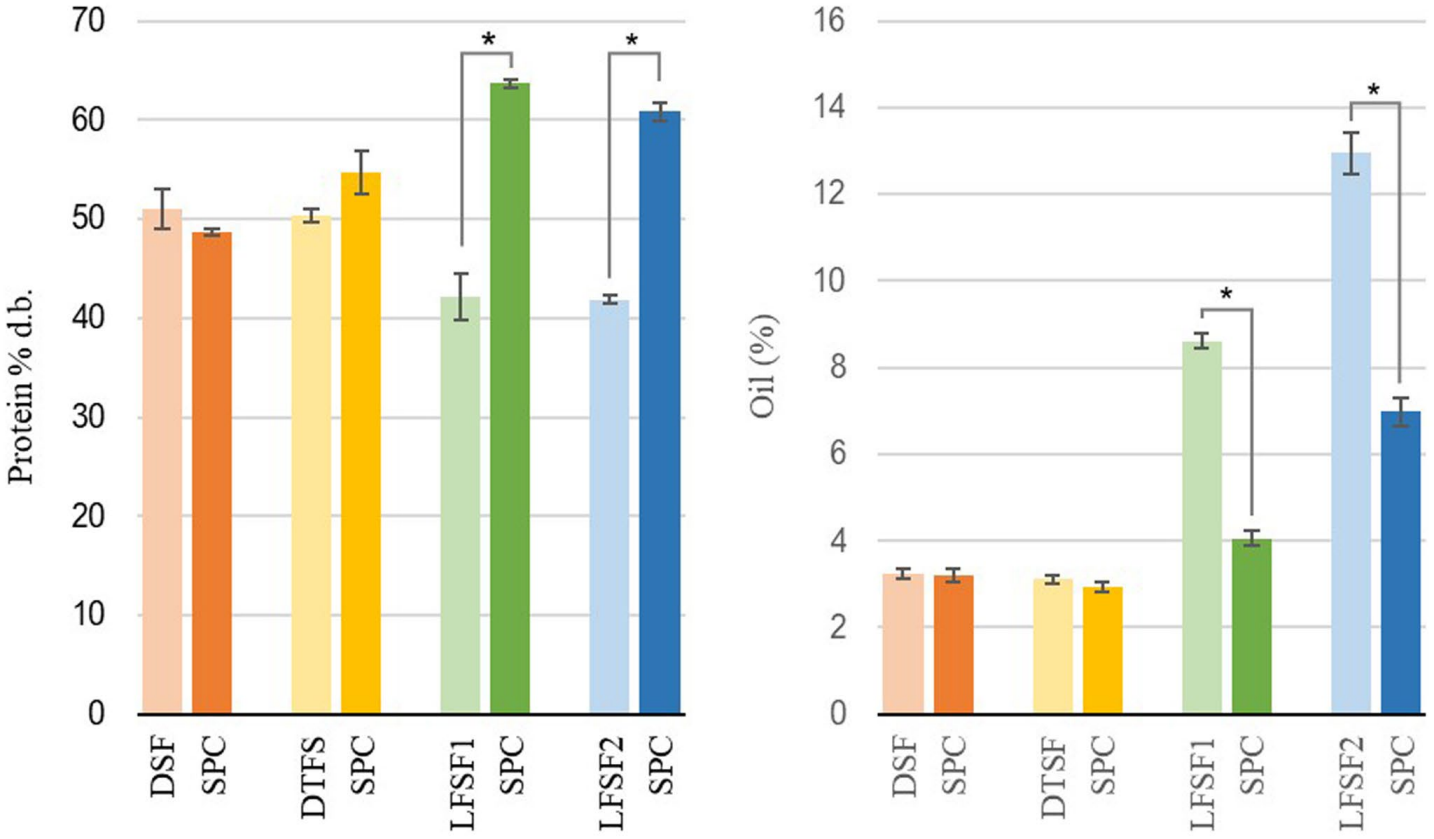
Total protein (left) and oil content (right) of raw materials and their soy protein concentrates (SPC) produced at lab scale (30 min, 22°C). Symbol (*) represents a statistical difference between the raw material and its SPC (Fisher’s LSD test; *p* < 0.05).

According to the obtained data, regardless of the processing parameters applied, the total protein content of SPC produced from DSF was not different than that of the starting material (*p* > 0.05). The SPC made from LFSF, however, had higher total protein content than their raw materials (*p* < 0.05; Figure 2). As shown in Figure 2, the total protein contents of SPCs made from DTFS, LFSF1 and LFSF2 samples were higher than that of their starting materials and reached up to 63.7% d.b. in SPC produced from LFSF1.

**Figure 3:**
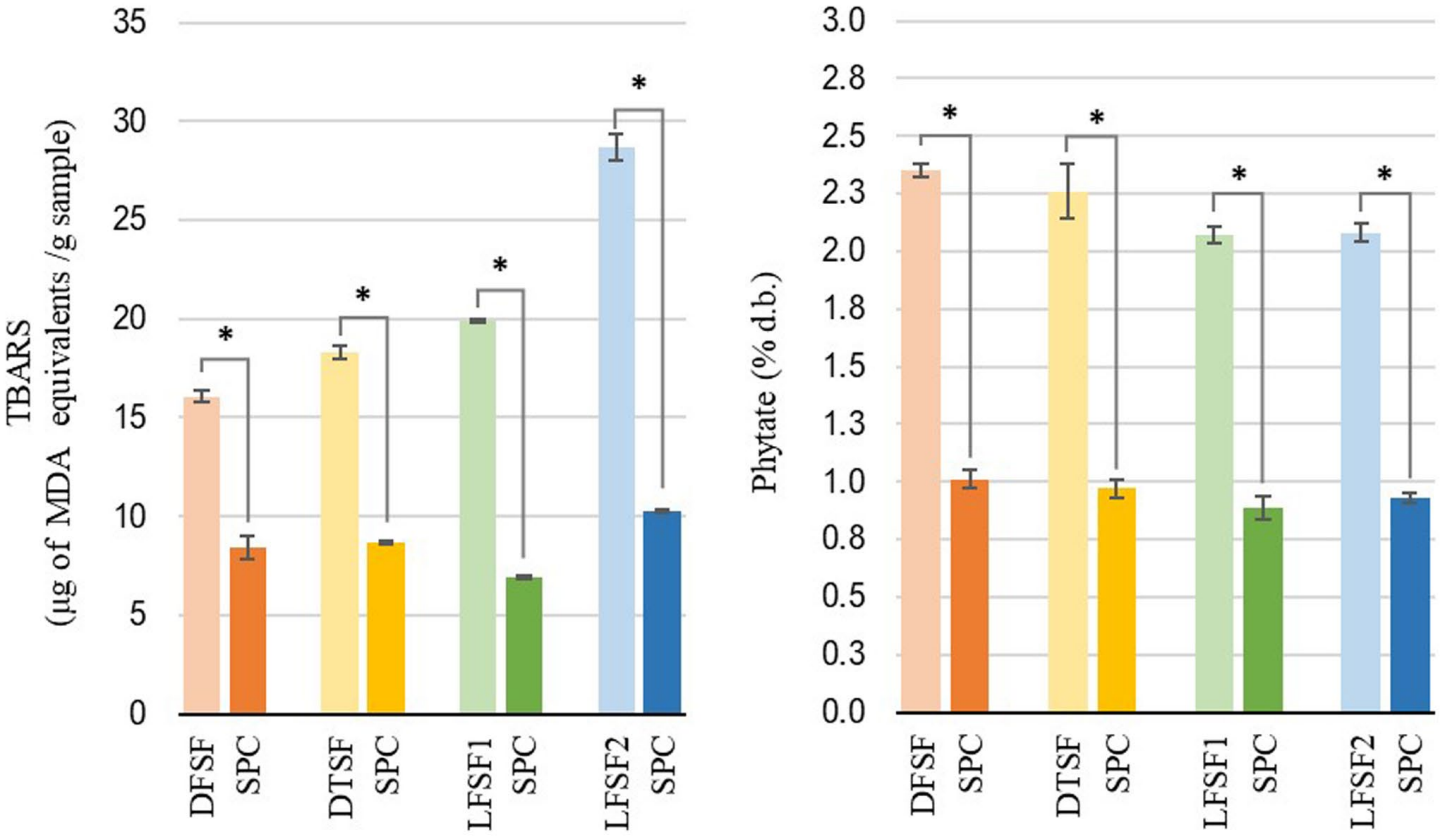
Thiobarbituric acid reactive substances (TBARS) measured as malondialdehyde equivalents (left) and total phytate content (right) of raw materials and their soy protein concentrates (SPCs) (30 min, 22°C) produced at the bench scale. Symbol (*) represents statistical difference between the raw material and its SPC (Fisher’s LSD test; *p* < 0.05).

The oil contents of DSF, DTSF, LFSF1, and LFSF2 were 3.23 ± 0.12% d.b., 3.11 ± 0.09% d.b., 8.60 ± 0.18% d.b., and 12.94 ± 0.46% d.b., respectively (Figure 2). The oil contents of SPCs obtained from LFSF1 and LFSF2 were 4.06 ± 0.18% d.b. and 6.98 ± 0.33% d.b., respectively, being 52.79 and 46.04% lower than their raw materials, respectively. To understand the impact of the alternative SPC production technique on the lipid oxidation degree of samples, the TBARS analysis was conducted on raw materials and their SPCs as it is a simple test indicating secondary oxidation of fatty acids due to their autoxidation into aldehydes and ketones (41). As shown in Figure 3, MDA concentrations across all samples decreased by more than 90% after the SPC production process (p < 0.05).

Despite their initial content, phytic acid in raw materials was reduced after SPC preparation. As shown in Figure 3, the preparation of SPC resulted in more than a 55% reduction in phytate. In raw materials, LFSF1 (2.06 ± 0.05% d.b.) and LFSF2 (2.08 ± 0.05% d.b.) samples had significantly lower phytate contents than DSF (2.35 ± 0.02% d.b.). After toasting, DTSF showed a lower phytate concentration than the DSF sample (Figure 3).

### 3.2. Proximate composition of SPC produced at the large scale

Three batches of SPC (SPC-1, SPC-2, SPC-3) were produced from LFSF1 with a final moisture content ranging between 8.6–9.8%. The SPC production yield and protein recoveries of the three batches were above 69.4 and 88.30%, respectively. The production yields were lower than the bench-scale production of SPC from LFSF1 (75.86%) indicating that more protein and non-protein compounds were lost in the large-scale trials (Table 2).

**Table 2:** SPC yield, protein recovery, and proximate composition of LFSF1 and three batches of its SPC at the large-scale production trials (30 min, 22°C).

	Moisture (%)	SPC yield (%)	Protein recovery (%)	Total protein (% d.b.)					Oil (% d.b.)				Crude fiber (% d.b.)				Ash (% d.b.)				Total carbohydrate (% d.b.)
LFSF1	4.56			48.20	±	0.09		b	8.00	±	0.88	a	5.71	±	0.87	a	5.89	±	0.02	a	37.91
SPC-1	9.77	71.18	91.10	61.68	±	0.34		a	4.81	±	0.17	b	9.97	±	1.67	b	4.20	±	0.02	b	29.31
SPC-2	7.65	69.49	88.30	61.25	±	0.10		a	4.60	±	0.07	b	9.07	±	0.17	ab	4.28	±	0.00	b	29.87
SPC-3	8.64	69.40	88.35	61.36	±	0.29	a	4.74	±	0.27		b	10.78	±	1.93	b	4.13	±	0.13	b	29.77

LFSF, low-fat soy flour; SPC, soy protein concentrate. ^a,b^Means with different superscript letters within each column parameter are statistically from each other, after One-Way ANOVA and the Fisher’s LSD post hoc test (*p* < 0.05).

The proximate compositions of LFSF1 and resulting SPCs are shown in Table 2. Each SPC batch was produced following the same protocol, using the same equipment and materials. It should be noted that protein, oil, crude fiber, and ash contents of the three SPC batches were not different from each other (*p* > 0.05). As observed in bench-scale trials, the total protein content of SPC (61.25–61.68% d.b.) was higher than that of the raw material (48.2 ± 0.09% d.b., *p* < 0.05). It is noteworthy that the total protein content of LFSF1 measured by the Bradford assay (42.13 ± 2.32% d.b.) was lower than the one calculated after the measurement with the CHN analyzer. One reason for this difference might be that the sonication process applied to break apart protein in the Bradford assay was not sufficient to disperse all proteins in the sample, which might underestimate the total protein content of the sample. The crude fiber of LFSF1 was also measured as 5.71 ± 0.87% d.b., which was in line with the value in the certificate of analysis (6.26% d.b.) provided by the manufacturer. In line with the bench-scale results, the oil content decreased by more than 39% after the preparation of SPC in all three batches (*p* < 0.05). Similar to the oil content, ash and total carbohydrate amounts in produced SPCs were lower than the LFSF1.

### 3.3. Lipid oxidation level of SPC produced at the large scale

The peroxide value in LFSF1 was 12.03 ± 0.97 mEq/kg oil (Table 3), which is acceptable according to the CODEX (47), established for cold-pressed and virgin oil at 15 mEq/kg oil. Upon processing into SPCs, the peroxide value was significantly reduced (*p* < 0.05). Similar trends were observed in MDA and hexanal evaluations, which were used to examine the presence of secondary lipid oxidation products. The initial MDA and hexanal concentrations of LFSF1 were measured as 18.33 ± 0.15 μg MDA equivalent/g sample and 1.00 ± 0.007 μg/g sample, respectively. In SPCs, both MDA and hexanal concentrations were lower than that of the LFSF1 (*p* < 0.05), showing a 64.8 and 34% reduction, respectively. The three batches of SPCs had similar values for the three oxidation parameters (Table 3).

**Table 3:** Peroxide value, malondialdehyde, and hexanal concentration of LFSF1 raw material and three batches of its SPCs at the large-scale production trials (30 min 22°C).

	PV (mEq/kg oil sample)				MDA (μg MDA equivalents/g sample)				Hexanal conct. (ppm)			
LFSF1	12.03	±	0.97	a	18.33	±	0.15	a	1.00	±	0.07	a
SPC-1	5.47	±	0.50	b	6.69	±	0.20	b	0.70	±	0.06	b
SPC-2	6.51	±	0.49	b	6.45	±	0.43	b	0.66	±	0.06	b
SPC-3	5.52	±	0.46	b	6.59	±	0.24	b	0.67	±	0.03	b

LFSF, low-fat soy flour; MDA, malondialdehyde; SPC, soy protein concentrate; PV, peroxide value. ^a,b^Means with a different superscript letter within the same column are statistically different from each other at *p* < 0.05.

### 3.4. *In-vitro* protein digestibility of SPC produced with low resource processing method

The amino acid composition of LFSF1, three SPCs produced from it and casein (the control) is presented in Table 4. Glutamic acid/glutamine was found in the highest concentration in both LFSF1 and SPCs. As expected, the digestibility of casein control was found as 100% (Table 5). The protein digestibility of LFSF1 was estimated at 91.1%. This value was increased significantly after processing into SPCs (*p* < 0.05). The three batches of SPCs produced from LFSF1 had an average *in vitro* protein digestibility of 95.1 ± 0.05%.

**Table 4:** Amino acid composition of LFSF1 and its SPC at the large-scale production trials.

	LFSF1	SPC 1	SPC 2	SPC 3	Casein control^a^
Non-essential AA (g/100 g sample as is basis)					
Aspartic acid	4.16	3.77	4.24	3.94	5.24
Serine	2.90	3.33	3.52	3.37	3.00
Glutamic Acid	6.09	5.60	6.59	6.68	16.14
Proline	1.81	2.06	1.80	1.96	7.61
Glycine	2.52	3.27	3.48	3.04	1.20
Alanine	2.13	2.45	2.47	2.40	2.01
Arginine	3.69	4.29	4.29	3.86	2.79
Cysteine	0.77	0.78	0.76	0.78	0.27
Tyrosine	1.43	1.79	1.66	1.98	4.50
Essential AA (g/100 g sample as is basis)					
Histidine	1.46	1.75	2.03	1.83	2.14
Isoleucine	1.71	1.92	1.90	1.93	4.01
Leucine	2.96	3.38	3.49	3.45	7.00
Lysine	3.75	4.61	4.62	3.97	2.28
Methionine	0.47	0.78	0.73	0.82	4.00
Phenylalanine	1.90	2.12	2.22	2.20	2.97
Threonine	1.76	2.25	1.96	1.98	6.34
Tryptophan	0.64	0.70	0.69	0.69	1.17
Valine	1.83	1.81	2.06	2.05	4.79

AA, amino acid; LFSF1, low-fat soy flour-1; SPC 1–3, three separate batches of soy protein concentrates

a Amino acid composition of the casein control was provided by the Megazyme™.

**Table 5:** Protein digestibility corrected amino acid score (PDCAAS) for LFSF1 and its SPCs at the large-scale production trials.

Essential amino acid (mg/g protein as is)		LFSF1	SPC 1	SPC 2	SPC 3	FAO/WHO amino acid reference pattern
Histidine		31.65	31.44	35.82	32.65	19
Isoleucine		37.14	34.41	33.55	34.35	28
Leucine		64.37	60.77	61.73	61.49	66
Lysine		81.57	82.81	81.63	70.81	58
Methionine + Cysteine		26.92	27.97	26.29	28.45	25
Phenylalanine + Tyrosine		72.34	70.24	68.54	74.67	63
Threonine		38.26	40.37	34.60	35.35	34
Tryptophan		13.91	12.58	12.20	12.31	11
Valine		39.88	32.57	36.40	36.65	35
*In Vitro* Protein Digestibility (IVPD) (%)	Casein control		95.2%	95.5%	94.6%	
	100 ± 0.001	91.1 ± 0.001	95.1 ± 0.005*^,^^a^			
AAS		0.975	0.921	0.935	0.932	
LEAA		Leucine	Leucine	Leucine	Leucine	
PDCAAS			0.88	0.89	0.88	
		0.89 ± 0.001	0.88 ± 0.01^n.s.^			

AAS, amino acid score; LFSF1, low-fat soy flour-1; LEAA, limited essential amino acid; SPC 1–3, three separate batches of soy protein concentrate. NS = no statistical significance.

* *p* < 0.05, determined by the student’s t-test, between the LFSF1 and SPC samples.

a The average in vitro protein digestibility value of three batches of SPC produced from LFSF1.

As shown in Table 5, the PDCAAS of LFSF1 was calculated as 0.89 and three batches of SPC produced from it also had a mean PDCAAS value of 0.88 ± 0.01 after the low-resource method was implemented (*p* < 0.05). Although the protein digestibility of SPCs was higher than that of LFSF1 because the limiting amino acid score was relatively lower in SPC, the PDCAAS values remained unchanged after processing.

## 4. Discussion

### 4.1. Evaluation of the effect of alternative method on protein, oil, phytic acid concentration, and oxidation level of SPC produced at the bench scale

The application of the low-resource, water-washing method on LFSF resulted in SPC having higher total protein content and lower oil, phytate, and oxidation levels. The increment in the total protein content after the processing is due to the removal of soluble and dispersible solids and oil into the water phase during the mixing process. Peng et al. (31) also observed that after the ethanol/water washing process of soy protein concentrate production, regardless of the type of solvent used, the total protein content in SPCs was enriched after the washing process and it was attributed to the removal of sugars and oil from the raw material during the mixing stage.

Moreover, the extrusion-expelling process applied to LFSF and toasting of DTFS also had an impact on the total protein content of their SPCs by causing heat-induced denaturation of proteins, which is less dispersible in the water phase and most remained in the precipitate during the low-resource processing, leading to increased total protein content in resulting SPCs. The level of heat denaturation has been correlated with the protein dispersibility index (PDI), which is an important parameter for protein functionality and an indirect indicator for the extensivity of heat treatment applied to materials. As the intensity of heat treatment increases, protein denaturation increases along with a decrease of the PDI (48). PDI is also correlated with protein solubility. Therefore, high-temperature treatments such as those applied during the production of LFSF and DTFS, reduce their PDIs and their solubilities. As a result, a larger amount of the protein fraction is unable to disperse in the water, increasing protein yield extracted after this alternative, low-resource SPC production method.

The increase in the total protein content can also be attributed to the partial dispersion of oil from the materials to the mixing solution. Peng et al. (31) prepared SPCs from cold-pressed soy meal by an aqueous ethanol washing method and investigated the effect of water/ethanol ratios on the overall composition and the functionality of resulting SPCs. They mixed cold pressed soy meal with ethanol/water solutions (1:10 w/v) prepared at different concentrations (0 to 100%) at 25°C for 30 min and centrifuged at 20,000 × *g* for 30 min. Their results indicated that when water was used as a solvent alone, the oil content decreased by about 2%. This reduction was lower than what was found in the present study, yet still lower than the original material. The observed difference in oil reduction upon processing might be due to the difference in raw material and actual process between the two studies. It has been reported that dry extrusion applied during an extrusion-expelling combined process disrupts the integrity of the cellular structure of soybean cotyledon, which facilitates oil release from subcellar structures. This extrusion process turns the soybean material into a more homogenous matrix at a semifluid state and the oil becomes readily available to press out at the screw-pressing step (49).

Reduction in phytic acid content can be explained by the high solubility of phytate in water (50). It is known that phytic acid is heat labile (51). Thus, the difference in phytate contents between DSF and the other three raw materials might be due to high temperatures applied during extrusion expelling and toasting processes. Clydesdale and Camire (52) also demonstrated that phytic acid content of defatted soy flour significantly reduced after toasting at 77°C for 1 h and boiling for 1-h applications. Many authors have also shown that extrusion can significantly decrease phytate concentrations in other cereals and legumes including lentils, beans, peas, corn, finger millet, rice, and wheat (53). This reduction could be explained by the degradation of this antinutrient by high temperature, shear forces and pressure levels applied during the extrusion process that leads to hydrolysis of inositol hexaphosphate (IP6) to lower molecular weight forms (54).

The water-washing process improved the oxidative stability of SPC prepared at the bench scale. One explanation for the reduction of MDA concentration might be that most oxidized oil might be removed from the samples with the water mixing process and secondary oxidation products like aldehydes are partially polar, diffusing easily into the aqueous fraction. However, as observed in the study of Papastergiadis et al., (41), due to the interference of other compounds found with 2-thiobarbituric acid (TBA), the TBARS test overestimates the content of MDA in some foods, like soybeans, corn, dry nuts and cheese. Therefore, to better evaluate the effect of alternative SPC production technique on the lipid oxidation degree of samples, other oxidative stability parameters should be tested. For that purpose, SPCs produced at a larger scale were evaluated not only for their MDA concentrations but also their peroxide values and hexanal concentrations as indicated below.

### 4.2. Evaluation of the low-resource SPC method on large-scale production trials

The large-scale trials confirmed the bench-scale findings in which applying the alternative water washing method, resulted in SPCs with higher protein (from 48.2% d.b. up to 61.7% d.b.) and fiber (from 5.71% d.b up to 10.8% d.b.) contents. At the same time, the contents of oil, total carbohydrates, ash, and phytate were lower than the starting material. Interestingly, this process resulted in a protein of higher digestibility and a material with improved oxidative stability (as indicated by reduced values of several oxidation markers).

The use of water without pH modification is currently not applied to recover proteins as in SPC. The type of solvent used in the washing process and starting material might lead to obtain lower yields and protein recoveries with the proposed washing method compared to previous studies. In the study of Wang et al. (55), higher recoveries of soy protein concentrates were obtained when it was produced from extruded-expelled soybean meals by using acid and alcohol wash methods (over 78% yield and more than 95% protein recovery). Peng et al. (31) also observed that using water alone as a solvent in the washing process resulted in the lowest yield (around 45%) and protein (approximately 50%) recovery, increasing the concentration of ethanol in the solvent up to 80% increased the yield and protein recovery to almost 81 and 90%, respectively. As indicated by Luthria et al., (56), particle size, the pre-treatment method applied to raw materials, the solid–liquid ratio during the solvent-washing process, and the solid–liquid separation method also have an impact on SPC production and protein yields. The yield recoveries found in this study for total SPC and protein, though lower than using alcohol, are technically feasible under the current processing conditions in SSA.

Crude fiber was also increased in SPC. Crude fiber is the insoluble part of dietary fiber in plant foods that remains after sequential extraction with diluted acid and alkaline solvents (57). Soaking or washing legumes or flour can lead to the release of some water-soluble compounds, including soluble fibers like pectin and some hemicellulose, but insoluble fiber remains in the material. Soybean meal produced from extrusion expelling (~8% oil) typically contains about 6% crude fiber. After the application of the proposed washing technique, the crude fiber contents were estimated at 10%. Wang et al. (55) used an alcohol-washing method for the preparation of SPC and reported an increase of crude fiber in SPCs made from white flakes (from 3.94 to 4.39%) and extruded expelled soybean meals (from 4.06 to 5.36%).

Soy protein concentrates typically have less than 1% lipid on a moisture-free basis when they are made from dehulled, defatted, solvent-extracted soybean meal and processed by the acid or alcohol wash methods (58). As we and others have shown, soybean meal obtained after the extrusion and expelling processes still contains a considerable amount of oil. Nelson et al. (17) reported that up to 59% of the oil in whole soybeans could be removed by the extrusion expelling process, leaving a soy cake with 6–9% oil. Similarly, Wijeratne et al. (49) reported that the amount of oil in low-fat soy cake after extruding expelling processing ranges between 6 to 9%. Despite its cost, it is possible to capture this oil washed away using a multiphase decanter centrifuge. The results showed that with the proposed alternative washing method, up to 42.5% of the oil remaining in a typical extruded expelled soy flour can be removed.

Ash and carbohydrate contents in SPC were lowered (from 5.9 to 4.2%) after the application of this processing method. The ash content of LFSF1 was similar to the total ash content (5.96%) of press cake obtained after extrusion expelling of whole soybeans (17). Ash is the inorganic residue that comprises of minerals left after either the combustion or complete acid-facilitated oxidation in a food material (59). Generally, soy protein concentrates contain an ash content between 3.8 and 6.2% d.b. (60). According to CODEX (61) for soy protein products, the yield of ash shall not exceed 8% on a dry weight basis. Total carbohydrate reduction in SPC is due to the partial release of water-soluble carbohydrates, such as some oligosaccharides and simple sugars, during the washing process (62). Carbohydrate content was similar to that reported by Nelson et al. (17), who calculated the total carbohydrate content of soy cake produced from extrusion expelling as 37.38% d.b. According to USDA’s Food Composition Database, soy protein concentrate produced by acid wash or alcohol extraction has around 27% total carbohydrate on a dry matter basis (63, 64). Wang et al. (65) also reported that soy protein concentrates produced from two different extruded expelled soybean cakes by using the alcohol washing method had lower total carbohydrate contents (15.78 and 17.02%) than their starting materials (23.26 and 24.01%).

The oxidation makers, MDA, hexanal, and peroxide value, which accumulated after the extrusion and expelling process were almost halved in SPC after water washing. MDA is a terminal product of lipid oxidation process and is a useful marker for the quantification of lipid peroxidation in foods. However, as mentioned earlier, the TBARS assay overestimates MDA concentration in some foods due to other interferences in the sample. It has been shown that hexanal is one of the important volatile compounds found in soybean and soybean products contributing to the beany flavor originating from lipid oxidation of polyunsaturated fatty acids (66, 67). Lei and Boatright (68) reported that among all the volatiles they detected in the headspace samples of SPC slurries (35 g SPC in 500 ml water), hexanal displayed the highest peak. Therefore, in addition to the MDA concentrations, the hexanal content of LFSF1 and its SPCs were also determined to obtain more information on their lipid oxidation status. Previous studies have shown that soybean lacking lipoxygenase enzymes or soy products prepared from lipoxygenase-free soybeans had less beany flavor and contained less hexanal and other volatile compounds than regular soybeans. Therefore, it has been postulated that one of the major contributors to the formation of hexanal and other carbonyl compounds in soy products was the activity of the lipoxygenase enzyme (LOX). Several studies have shown that heat treatments are effective in inactivating LOX isoenzymes in soybeans (69–72). Crowe et al. (73) examined the effect of the extrusion/expelling process on the soybean lipoxygenases and found all three isoenzymes were 100% inactivated in partially defatted soy flours extruded at 117°C and higher barrel temperatures. The extrusion/expelling process applied in the production of LFSF1 might have caused the inactivation of LOX and led to the formation of less hexanal in the sample. With the proposed water washing method, the hexanal and other secondary products of lipid oxidation, like MDA, might be further removed by releasing into the mixing water along with the oil removed from the sample. Altogether the evidence presented shows that the water-washing method can enhance the oxidative stability of SPC.

Protein digestibility increased by 4 percentage points in SPC, which influences its final protein quality. Both LFSF1 and its SPCs can be considered complete proteins, whose amino acid profile was close to the reference profile as established by the FAO/WHO, except for leucine which was the limiting amino acid. As shown by others, soybeans are high in glutamic acid (74, 75). The amino acid score (AAS), which is the ratio of the amino acid content in 1 g of a target protein to that of a reference protein or requirement, of leucine of all samples ranged from 0.92 to 0.98 (Table 5). Despite ample evidence suggests sulfur-containing amino acids, more specifically methionine, are the dominating limiting amino acids in legumes, other branched-chain amino acids, including leucine, have also been reported as limiting in previous studies (76–80). The variations in essential amino acid composition and limiting amino acids of cereals and legumes might be due to differences in crops’ genetic background, the total protein content of the original cultivar., the processing methods applied, as well as the nature of amino acid biosynthesis pathways (81–83).

Processing modifies protein digestibility, and similarly, protein digestibility modifies protein quality, especially in vegetable proteins (84). Protein digestibility of LFSF1 was higher than previously reported [71.8% in (85) and 75.3% in (86)]. The extrusion expelling process applied in the production of LFSF1 material might lead to higher digestibility values than the results reported in previous studies due to the elimination of its antinutrients. According to Hettiarachchy and Kalapathy (23), the protein digestibility of full-fat soy flour and defatted soy flour are in the range of 75–92% and 84–90%, respectively. Thus, the values in this study are not at all unexpected. Soybeans contain a variety of potential antinutrients including phytates, saponins, protease inhibitors, isoflavones, lectins, oligosaccharides, and tannins. These antinutritional factors have a detrimental effect on the nutritional value, utilization, and digestibility of soybean protein (23, 87). A wide range of processing methods can improve the digestibility of proteins in legumes by reducing or eliminating these antinutritional factors (88–90). A high-temperature application during the extrusion process can effectively reduce antinutritional factors in legumes and their nutritional quality can be improved (53). For instance, Fasina et al., (91) indicated that trypsin inhibitor and lectin concentrations in soybean meal reduced from 34 and 2.25 mg/g meal to 5.52 and 0 mg/g, respectively, after extrusion expelling processing. Similarly, Marsman (92) observed that the trypsin inhibitor activity in untreated soybean meal became almost zero after extrusion at above 110°C resulting in an increase in the digestibility of the material by almost 80%.

While our results on protein digestibility were comparable to those reported for SPC by (77) (96.97%), altogether these were higher than those presented by Mohamed, et al. (93) and Obulesu and Bhagya (94), where the *in vitro* protein digestibility of SPC were 84 and 88%, respectively. This might be due to a difference in the raw material that was used to prepare SPC and the processing method applied. Obulesu and Bhagya (94) indicated that the SPC was produced by thermal processing of the defatted soy flakes followed by aqueous leaching and drying processes and the digestibility of the initial soy flour material used was 80%. The increase in the protein digestibility in the present study can be explained by the reduction in the phytic acid content. According to several authors, phytates can inhibit the activity of enzymes that are required for protein digestion (pepsin, trypsin, chymotrypsin) in the stomach and small intestine (95, 96), therefore their reduction can facilitate improvement of protein digestibility (88).

The PDCAAS values were lower than the results presented for soybeans and soy products (0.92–1.00) in previous studies (77, 97, 98). The reduction in the amino acid scores of limiting amino acid can be explained by the decrease in the concentration of leucine in the material after wash. As shown in Table 4, the concentrations for most amino acids were lower in SPC compared to the initial material. A large proportion of the protein was dispersed and lost during the wash, which might have influenced the final amino acid profile. A decrease in amino acid concentration after heat treatments was reported in previous studies, and this decrease was linked to the participation of amino acids in Maillard reactions (99–101). Drying temperatures after washing might have affected the amino acid profile as well (102), however, this was not empirically tested in this study.

## 5. Conclusion

The present report describes a simple process to produce SPC. It consisted of two major steps: (1) to extrude and expel soybeans into soy cake and grind the cake into low-fat soy flour, and (2) to produce SPC from the flour by extracting it with water and removing soluble components like phytic acid and soluble carbohydrates. Therefore, the process is characterized as low-resource, simple, and inexpensive. The low-resource method resulted in an SPC with higher total protein content, higher protein digestibility, higher crude fiber, and higher oxidative stability, while significantly decreasing phytic acid content. Compared to low-fat soy cake (flour), this high protein ingredient is significantly improved in many aspects and can be used to fortify basic staple dishes in the SSA region and address protein gaps in growing children, thus committing to SDG 2 targets. Further work includes investigation of the effect of low-fat soy cake handling on SPC quality and incorporation of SPC in various types of local foods and consumer acceptance.

## Data availability statement

The raw data supporting the conclusions of this article will be made available by the authors, without undue reservation.

## Author contributions

KL developed the proposed alternative processing method. EG, KL, KN and JA conceptualized the study. EG was primarily responsible for the design, execution, data collection, and analysis. JA critically assisted with all aspects of design, execution, and analysis. EG, AD, and JA drafted and revised the first draft. All the authors critically reviewed and revised the manuscript. All authors contributed to the article and approved the submitted version.

## Funding

This research was funded in part by the United States Agency for International Development (USAID) Feed the Future Innovation Lab for Soybean Value Chain Research, grant number AID-OAA-L-140000-1. This work was supported in part by funds from the Office of International Programs, College of Agricultural, Consumer and Environmental Sciences, University of Illinois at Urbana-Champaign.

## Conflict of interest

The authors declare that the research was conducted in the absence of any commercial or financial relationships that could be construed as a potential conflict of interest.

## Publisher’s note

All claims expressed in this article are solely those of the authors and do not necessarily represent those of their affiliated organizations, or those of the publisher, the editors and the reviewers. Any product that may be evaluated in this article, or claim that may be made by its manufacturer, is not guaranteed or endorsed by the publisher.
